# Superionic Conduction in the Plastic Crystal Polymorph of Na_4_P_2_S_6_

**DOI:** 10.1021/acsenergylett.1c02815

**Published:** 2022-03-22

**Authors:** Tanja Scholz, Christian Schneider, Maxwell W. Terban, Zeyu Deng, Roland Eger, Martin Etter, Robert E. Dinnebier, Pieremanuele Canepa, Bettina V. Lotsch

**Affiliations:** † Max Planck Institute for Solid State Research, Heisenbergstraße 1, 70569 Stuttgart, Germany; ‡ Department of Materials Science and Engineering, National University of Singapore, 9 Engineering Drive 1, 117575 Singapore; § Deutsches Elektronensynchrotron (DESY), Notkestraße 85, 22607 Hamburg, Germany; ∥ LMU Munich, Butenandtstraße 5-13, 81377 Munich, Germany; ⊥ Department of Chemical and Biomolecular Engineering, National University of Singapore, Engineering Drive 4, 117585 Singapore

## Abstract

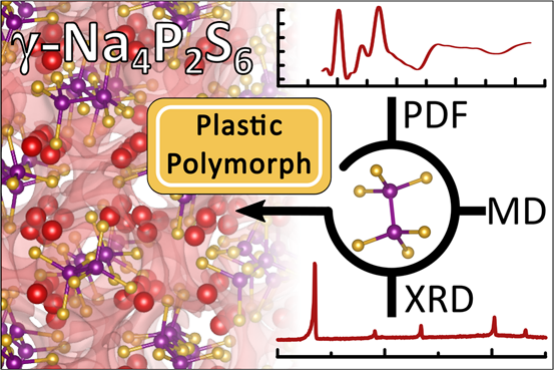

Sodium thiophosphates are promising materials for large-scale energy storage applications benefiting from high ionic conductivities and the geopolitical abundance of the elements. A representative of this class is Na_4_P_2_S_6_, which currently shows two known polymorphs−α and β. This work describes a third polymorph of Na_4_P_2_S_6_, γ, that forms above 580 °C, exhibits fast-ion conduction with low activation energy, and is mechanically soft. Based on high-temperature diffraction, pair distribution function analysis, thermal analysis, impedance spectroscopy, and *ab initio* molecular dynamics calculations, the γ-Na_4_P_2_S_6_ phase is identified to be a plastic crystal characterized by dynamic orientational disorder of the P_2_S_6_^4–^ anions translationally fixed on a body-centered cubic lattice. The prospect of stabilizing plastic crystals at operating temperatures of solid-state batteries, with benefits from their high ionic conductivities and mechanical properties, could have a strong impact in the field of solid-state battery research.

The rich chemistry of thiophosphates is an excellent platform for exploring compounds with interesting and functional properties. The plethora of possible thiophosphate anions ranges from the tetrahedral ortho-thiophospate PS_4_^3–^ to larger units, such as corner-sharing P_2_S_7_^4–^ or P_2_S_6_^4–^ having a P–P central bond. The ability of sulfur to form disulfur -S–S- bridges and the broad range of oxidation states of phosphorus allow for even larger, ring-like or square-shaped anions.^1,2^

Many thiophosphate compounds containing alkaline metals are ion conductors. However, the number of highly conducting phases is still limited to a few well-known representatives (e.g., Na_3_PS_4_). The as-prepared tetragonal (low-temperature) structure of Na_3_PS_4_ shows a low ionic conductivity of around 4.2 × 10^–6^ S cm^–1^ (50 °C), but it can be improved up to ∼4.6 × 10^–4^ S cm^–1^ (room temperature) by stabilizing the high-temperature cubic phase as a glass–ceramic.^3−5^ This was achieved by ball-milling the starting materials and subsequently annealing the glass at a low temperature of 270 °C to precipitate the cubic phase. Recently, Krauskopf *et al.* have suggested that the room-temperature stabilized cubic phase comprises tetragonal-like local-structure motifs.^6^

Another example of the phase-space flexibility of thiophosphates is Na_4_P_2_S_6_.^7,8^ This material can be synthesized *via* a precipitation route, as well as by high-temperature solid-state synthesis.^9,10^ Depending on the preparation method, Na_4_P_2_S_6_ crystallizes in the monoclinic polymorphs α- and β-Na_4_P_2_S_6_.^10,11^ These polymorphs can be transformed into each other by heating or annealing the sample. In this study, we identify a new high-temperature polymorph of Na_4_P_2_S_6_, γ, which displays plastic crystal characteristics.

Plastic crystals are characterized by a high degree of reorientational freedom, often found for spherical molecules, or conformational freedom, often found for polymers, of a cationic, anionic, or neutral sublattice of a solid.^12−14^ The disordered species are translationally fixed, therefore maintaining the lattice. This renders the material a solid with weak interactions in the partly “molten” anion sublattice. A number of inorganic ion conductors show these characteristics, including ABH_4_ (A = Li, Na, K, Rb, Cs),^15−17^ Li_2_SO_4_,^18,19^ LiAgSO_4_,^20^ LiNaSO_4_,^21^ Li_4_Zn(SO_4_)_3_^21^ Na_3_PO_4_,^22,23^ Na_2_AC_60_ (A = K, Rb, Cs),^24−26^ Na_2_B_12_H_12_,^27−29^ Na_2_B_10_H_10_,^30^ ACB_11_H_12_ (A = Li, Na),^31^ Li_2_B_12_H_12_,^29^ Rb_2_B_10_H_10_,^32^ Na_3_PS_4_,^33^ and Na_4_Zn(PO_4_)_2_^34^ (sorted by publication year). All of these examples contain rather globular-shaped anions with high point-group symmetries, such as *I*_*h*_ (e.g., B_12_H_12_^2–^ and C_60_^3–^), *D*_4*d*_ (e.g., B_10_H_10_^2–^), or *T*_*d*_ (e.g., SO_4_^2–^). High intrinsic symmetry of the anion species lowers the sterical hindrance for fast reorientational motion.^35^

The implementation of fast sodium-ion conductors in solid-state batteries is vital for large-scale applications such as power-grid regulation and short-term wind or solar energy storage. Cost and resource analyses have shown that using cheaper current collectors and cathode materials can reduce the price and weight of Na-ion (solid-state) batteries compared to their Li analogs, especially if prices of relevant resources such as copper and cobalt continue to rise.^36^ The more homogeneous distribution of Na deposits and the higher natural abundance (2.36% Na versus 0.002% Li)^37^ point to a sodium-driven future in grid (solid-state) battery technology. Solid-state batteries require among other properties high intrinsic ion conductivity and a robust mechanical interface between the ion conductor and the electrodes to provide high performance and long cycle life. Plastic ion conductor materials can beneficially provide weaker cation–anion interactions and increased volumes that allow for easier ion transport. Furthermore, the ductility of the plastic phase can help to maintain better contact with the electrode and reduce physical degradation of the interface during cycling. A major caveat is that plastic phase transitions in ion conductors are often observed at high temperatures, which impedes their application in real devices. Thus, it is vital to better understand the nature and formation behavior of plastic crystals to inform strategies for rationally lowering the transition temperature toward ambient conditions.

Here, we discover the new γ polymorph of Na_4_P_2_S_6_, which exhibits fast Na^+^ conduction with a low activation energy, a cubic crystal structure, and plastic crystal characteristics. The plastic phase transition is driven by the reorientational freedom of the elongated thiophosphate anion P_2_S_6_^4–^, rendering Na_4_P_2_S_6_ the first example of a salt, based on a prolate, complex anion, showing plastic crystal behavior.

Variable-temperature powder X-ray diffraction (PXRD) experiments were performed on Na_4_P_2_S_6_ well above the α–β phase transition at 160 °C.^11^ A new set of Bragg peaks is observed to emerge at 580 °C, as shown in Figure 1. The diffraction peaks of β-Na_4_P_2_S_6_ and the new crystalline phase coexist up to 585 °C (measured every 5 K), at which point the β phase disappears. This suggests the existence of a third polymorph of Na_4_P_2_S_6_, which we refer to as γ-Na_4_P_2_S_6_. Upon cooling, the transition is reversible with the re-formation of β- and α-Na_4_P_2_S_6_.

**Figure 1:**
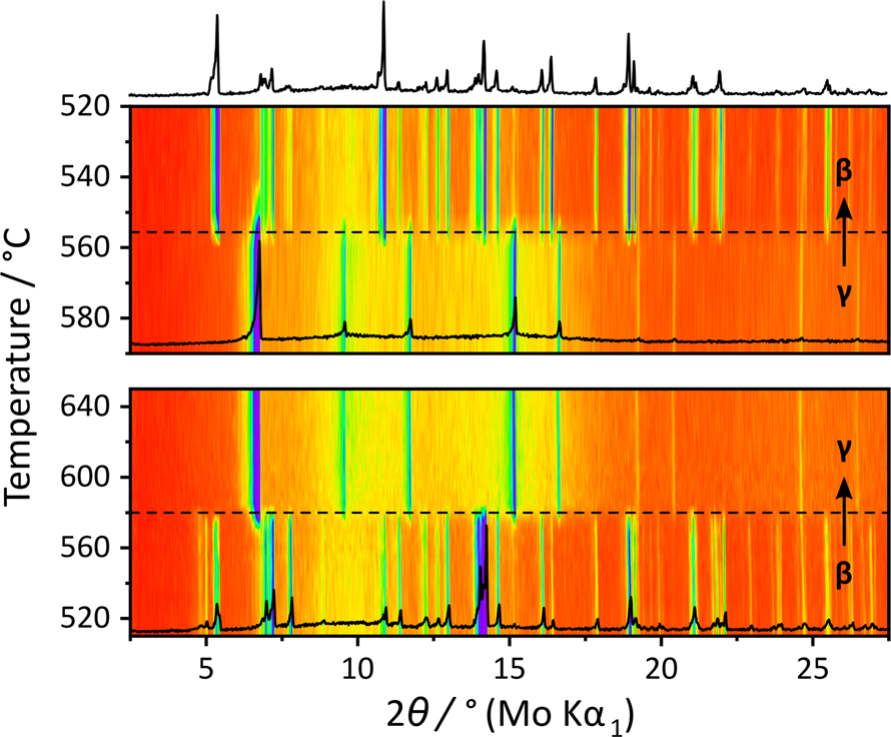
Variable-temperature PXRD of Na_4_P_2_S_6_ upon heating (bottom) and cooling (top) across the β–γ phase transition: contour plot of diffractograms (the color code, red to purple/blue, indicates the ranges from low to high diffraction intensities, respectively) and exemplary diffractograms (black) at 520 °C (β), 620 °C (γ), and 520 °C (β after heating above the phase transition). The selected diffractograms are additionally depicted in Supporting Information Figure S1.

Several remarkable features of the PXRD patterns of γ-Na_4_P_2_S_6_ are discussed in the following (labeled i–iv). These points lead us to conclude that γ is a newly observed member of the plastic crystal family.

(i) The Bragg peaks of γ-Na_4_P_2_S_6_ can be indexed to the cubic space group *Im*3̅*m* (No. 229) with a lattice parameter of 8.4509(3) Å at 580 °C. However, this high symmetry is not compatible with the ethane-like *D*_3*d*_ configuration of the P_2_S_6_^4–^ anions, suggesting that the anions cannot be crystallographically oriented in the structure. This observation suggests that some kind of static or dynamic disorder is present in the anion orientations.
(ii) The β–γ phase transition is accompanied by a large increase in diffuse scattering intensities, and the observed Bragg peak intensities rapidly decrease and disappear at higher angles. The diffuse scattering can be due to significant disorder, which could result for instance from different anion orientations, and lead to the destructive interference of higher order diffraction components. These are common features observable in the plastic crystal materials mentioned above.
(iii) The β to γ phase transition is accompanied by an unusually high increase in the volume of the unit cell, from 575.4 to 603.8 Å^3^/f.u. (assuming *Z* = 2 for γ). This gives an increase of ∼4.9% (see Figure 2a), which compares well to, e.g., ∼3.2% for the monoclinic to cubic transition in Li_2_SO_4_ and ∼10% for the cubic to orthorhombic transition in Na_3_PS_4_.^18,33,38^
(iv) A considerable hysteresis of ∼30 K in the β–γ transformation is observed from the diffraction data between heating and cooling, comparable to 40 K in Na_3_PS_4_.^33^

It is worth noting that, after cooling γ-Na_4_P_2_S_6_, changes in relative peak intensities are sometimes observed for the subsequent β and α phases (see Figure 1 and ESI). Similar behavior reported for γ-Na_3_PS_4_ was attributed to the development of preferred orientation.^33^ However, as no clear preferential orientations could be identified, and the effect was not observed in other experiments where the sample spent less time at elevated temperature (e.g., Figure S2), possible effects due to reaction or degradation of the sample cannot be excluded.

**Figure 2:**
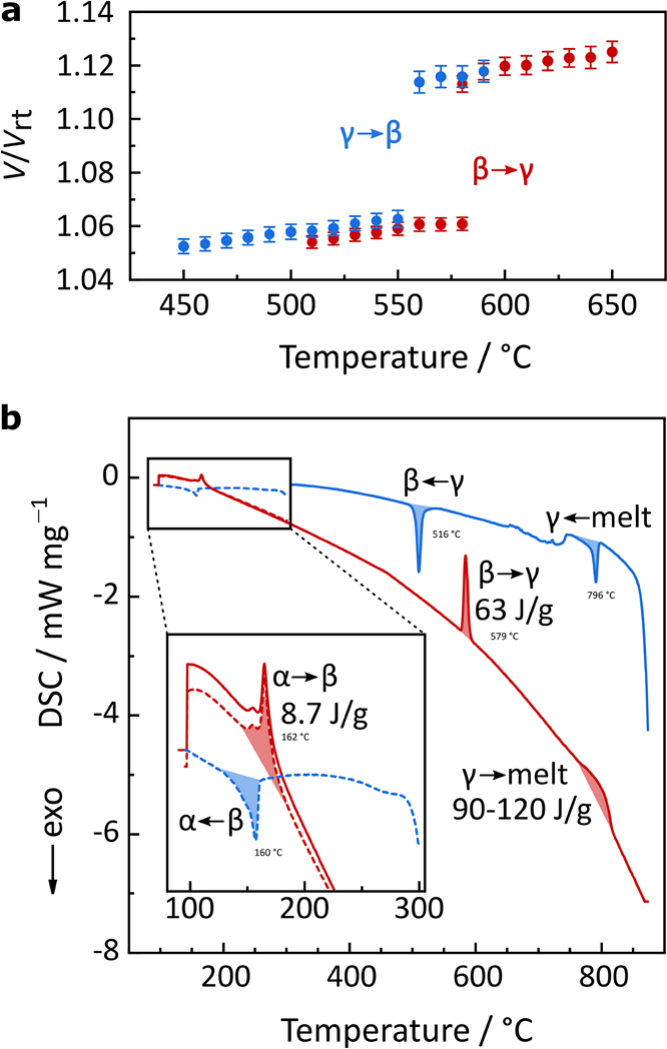
(a) Temperature-dependent volume expansion and contraction across the β → γ phase transition relative to the room-temperature unit cell volume, *V*_rt_, of Na_4_P_2_S_6_. (b) Differential scanning calorimetry (DSC) curve.

The phase transitions in Na_4_P_2_S_6_ were additionally studied with differential scanning calorimetry (DSC) as shown in Figure 2b, using a much faster heating rate (20 K min^–1^) than in the diffraction experiments. Overall, the DSC data confirm all three transitions α → β (160 °C), β → γ (580 °C), and γ to the melt (∼800 °C), and back. Additional PXRD patterns of α-, β-, γ- and molten-Na_4_P_2_S_6_ are displayed in Supporting Information Figure S2. The α → β phase transition involves very small atomic rearrangements and shows a correspondingly small latent heat of ∼8.7 J/g.^11^ In contrast, the β → γ transition has a latent heat corresponding to about half that of the melting transition (∼60 versus ∼90–120 J/g). This implies that substantial structural rearrangements are now required to form the γ phase. Other known plastic phase transitions also exhibit latent heats of similar magnitude, for example, 214 J/g in Li_2_SO_4_ or 150 J/g in Na_3_PS_4_.^18,33^ While the β → γ phase transition is observed at the same temperature as in the diffraction experiments (580 °C), the hysteresis on cooling appears much larger, ∼60 K. This could suggest kinetic limitations for the transformation back to the β phase.

To check if the P_2_S_6_^4–^ anion undergoes any structural change during the β → γ transition, we measured high-temperature Raman spectra. The spectra of Figure S3 display no change in the local *D*_3*h*_ anion symmetry from β → γ-Na_4_P_2_S_6_, supporting the assumption of an intact P_2_S_6_^4–^ unit at high temperature.

A direct structural solution of the γ-phase from the PXRD pattern is complicated by the incompatibility between the observed space group symmetry and the lower symmetry of the P_2_S_6_^4–^ anions. Therefore, we turned to the pair distribution function (PDF) analysis^39,40^ of synchrotron diffraction data to determine possible configurations to describe the local structure. Figure 3a shows the experimental PDF of γ-Na_4_P_2_S_6_ at ∼650 °C. Sharp peaks observed at 2.048 and 3.392 Å can be assigned to P–P/P–S and S–S (i–iii) atom-pair distances within the P_2_S_6_^4–^ anion. Another sharp peak at 2.801 Å can be assigned to the nearest-neighbor Na–S atom pairs. The peaks expected to correspond to pairs of S atoms located at opposite ends of the anion are not distinctly observed, which may suggest some flexibilty of the anion dihedral angle.

**Figure 3:**
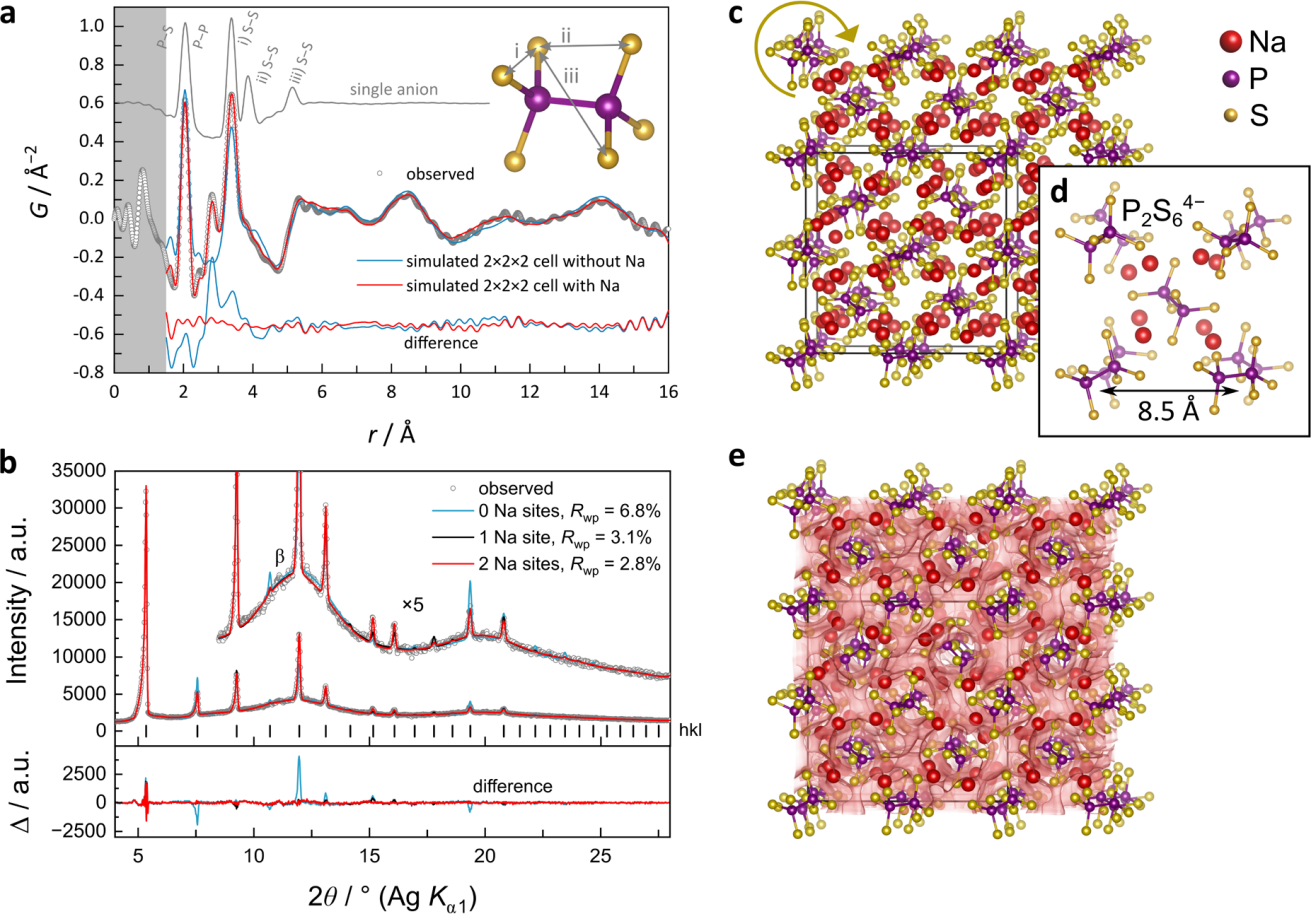
(a) Experimental synchrotron PDF with PDFs simulated from the fitted structure models and P_2_S_6_^4–^ anion only and (b) Rietveld fit of the PXRD pattern for γ-Na_4_P_2_S_6_ at 650 °C. The gray shaded region in (a) represents the unphysical interatomic distance range that is more highly affected by systematic errors from data processing. (c) Illustration of the PDF-refined γ-structure with a static approximation of the P_2_S_6_^4–^ anions in a 2 × 2 × 2 cell, and (d) in a single unit cell. (e) Overlay of bond valence energy landscape (red surface) and the crystal structure visualizing the three-dimensional Na^+^ conducting pathways.

Above 6 Å, the features in the PDF are broad and low intensity, in contrast to the PDFs measured for the α and β phases (see SI Figure S4). In line with the body-centered cubic (bcc) space group from indexing, a pseudocubic model was constructed, with *P*1 space group symmetry, in which the P_2_S_6_^4–^ anion barycenters are translationally fixed on the lattice points. The rotational orientations about the lattice points were allowed to refine freely against the experimental PDF data. A single-cell model (*Z*′ = 2) gives good agreement to short-range distances and indicates that neighboring anions prefer not to align in the same orientation. However, the model fails when *r* is greater than the lattice parameter due to the fixed geometry of the second nearest neighbor anions. A 2 × 2 × 2 supercell (*Z*′ = 16) was further constructed to test against the data at longer distances. This model allows for a much larger distribution of relative anion orientations sampled at any given distance and also showed good agreement to the data up to approximately 16 Å. Overall, the data at higher distances prefer models with more orientational disorder. This suggests weak or no correlation between relative anion orientations at long distances (see SI Figure S5).

In order to generate a complete model that is compatible with the data, 8 Na sites were introduced to the single-cell model (64 Na sites to the supercell model) and allowed to refine with restraints set for the distances of closest approach: 2.5 Å (Na–S), 3.4 Å (Na–P), and 3.5 Å (Na–Na). A unique set of positions could not be determined, but the refinements preferred the Na atoms to distribute throughout the “interstitial” space around the structure-forming anions, optimizing to give the best agreement to the preferred Na–S neighbor distance. The good agreement between simulated and experimental PDFs and resulting structure are shown in Figures 3a,c,d.

Inspired by the real-space model, Rietveld refinements were performed using the space group *Im*3̅*m* with a single anion fixed at the origin, giving 48 anions each at the corner and body-center position (mirroring free rotation of the anions averaged over all unit cells). Crystallographic data and refinement details, as well as atomic positions and displacement parameters, are compiled in Tables S1 and S2 in the Supporting Information. The occupancy was fixed to . Then Na atom sites were sequentially added and refined until the Bragg peaks could be reproduced. This resulted in two symmetry-independent sites giving 96 positions each, with the occupancies constrained to give the expected stoichiometry (details in the Experimental Section). Similar to the model from PDF refinement, the Na atoms again distribute to fill the “interstitial” space around the structure-forming anions. The Rietveld fit is shown in Figure 3b. By comparison of the *Im*3̅*m* model to higher distances in real space (see SI Figure S5), it is found to accurately reproduce the on-average pair-density fluctuations beyond ∼8 Å. For the real-space refinements, it was necessary to set up the models with separate intra- and intermolecular atomic displacement parameters to properly describe the correlated motion.^41^ To test for disorder within the anions, separate parameters were defined to describe the peak widths of different atom-pair types.^42^ We found that correlations between opposing S–S pairs were broader than other atom-pair contributions by a factor of more than 2, which further supports the hypothesis of torsional distortions (along the P–P bond) of the anions.

In a complementary approach to locate and count low-energy sites and possible migration pathways for Na^+^, softBV (bond valence) analysis^43,44^ was conducted for the PDF-derived static approximation in a 2 × 2 × 2 cell. Figure 3e illustrates the BV landscape and reveals many of the possible interstitials that exceed the number of Na^+^ by far. Therefore, the crystal structure offers a high number of vacancies, which are beneficial for fast-ion conduction. A large number of alternative pathways can be found in this structure with energy barriers of comparable barrier heights of ∼230 meV.

After obtaining insights into the ion transport pathways of γ-Na_4_P_2_S_6_ via BV modeling, an experimental characterization was attempted. High-temperature electrochemical impedance spectroscopy (EIS) measurements were performed on pellets of Na_4_P_2_S_6_. The malleability of γ-Na_4_P_2_S_6_ resulted in pellets being deformed under the slightest contact pressure, changing their thickness as a result, and even leaking out of the setup. Additionally, the chemically aggressive nature of Na_4_P_2_S_6_ had major implications on the corrosion of electrodes and contacts within a short amount of time. Contrary to good EIS measuring practice, the data shown here were obtained with low contact pressures on the pellets, short equilibration time, and low-temperature-step density, but, nevertheless, highlight the characteristics of the phase transition. The results of the measurement are presented in the Arrhenius plot of Figure 4a (see SI Figure S8 for exemplary spectra). For β-Na_4_P_2_S_6_ (520–578 °C), an activation energy of 562(48) meV was found, which is slightly higher than the previously reported value obtained in measurements at lower temperature.^11^ The β → γ transition at around 580 °C causes an abrupt increase in the ionic conductivity from 92(11) to 140(12) mS cm^–1^. The order of magnitude in ionic conductivities for β- and γ-Na_4_P_2_S_6_ is comparable to Na_3_PS_4_, where the β → γ transition at 500 °C is accompanied by a jump from ∼40 to ∼400 mS cm^–1^.^33^ γ-Na_4_P_2_S_6_ exhibits a lower activation energy of about 89(18) meV, much lower than the BV calculated value on a rigid model, but comparable to γ-Na_3_PS_4_ (110(10) meV).^33^ The behavior of ionic conduction is reversible on cooling, with a similar hysteresis of ∼40 K as found in the diffraction experiments. The extracted ionic conductivities upon cooling the pellet are higher than for heating. This unusual behavior can be explained by accelerated decomposition of Na_4_P_2_S_6_ at elevated temperatures. The visual appearance of the pellet changed from initially white to dark gray after cooling. The aggressive nature of Na_4_P_2_S_6_ at elevated temperatures is reflected in the corrosion of the platinum electrodes after the EIS measurement (see images in Figure S7). Presumably, Na_4_P_2_S_6_ reacts with the Pt electrodes and forms some electronically conducting phases that increase the conductivity, leading to apparent lower activation energies compared to the heating cycle. Hence, the extraction of a true activation energy for ionic motion is vitiated for the cooling cycle. We suspect the loss of sulfur and the reaction of sodium ions with ceramic or quartz glass parts of our setups to be the origin of the accelerated decomposition of γ-Na_4_P_2_S_6_ that we observed in some experiments. We refer to the Supporting Informaiton for more details. Extrapolating the linear Arrhenius behavior of γ-Na_4_P_2_S_6_ to 25 °C results in a room-temperature ionic conductivity of 50(24) mS cm^–1^, similar to the findings of Famprikis *et al.* for Na_3_PS_4_.^33^ Despite our efforts to rapidly quench Na_4_P_2_S_6_ from high temperature by submerging an ampoule in ice water, we were not successful in stabilizing the cubic γ phase at room temperature.

**Figure 4:**
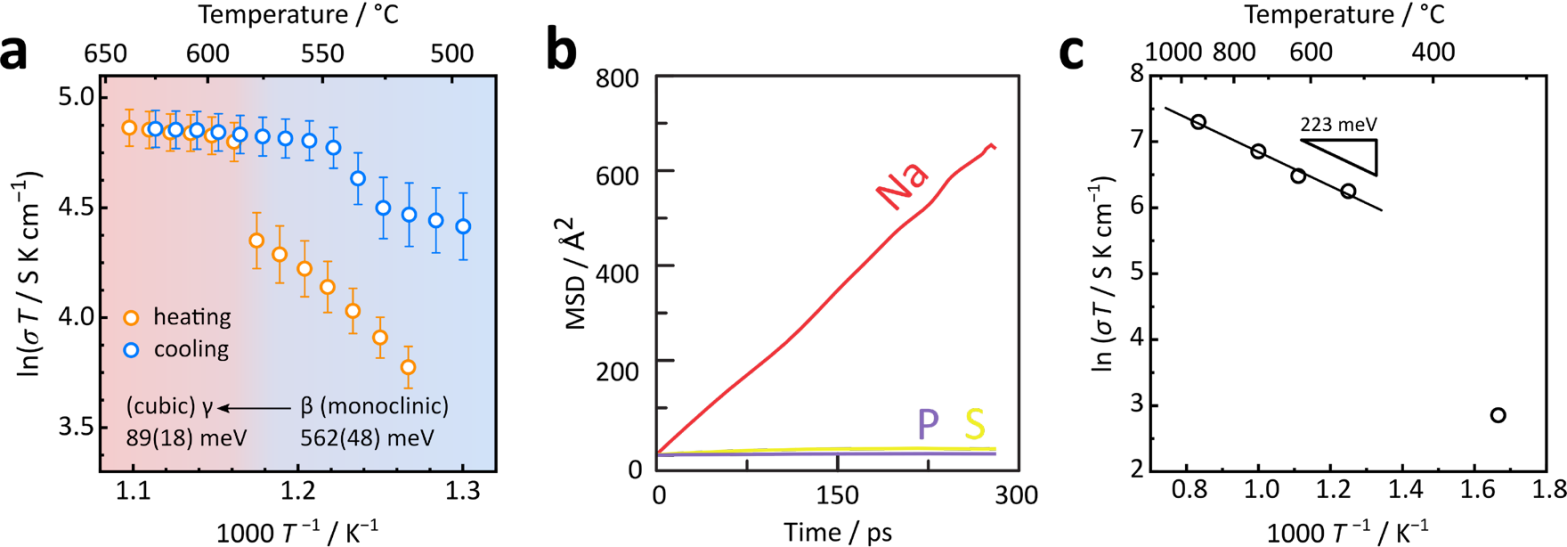
(a) Arrhenius plot of ionic conductivity and activation energies derived from impedance spectroscopy. The heating and cooling segments are indicated by orange and blue circles. The activation energy was extracted from the orange (heating) data points, since the cooling data points possibly describe a mixed ionic–electronic conducting (MIEC) phase. The error bars arise from the standard deviation of ionic conductivity measured on three distinct pellets. (b) Mean-squared displacements (MSDs) of Na, P, and S at 1000 K from AIMD simulations. (c) Arrhenius plot of the ionic conductivity and activation energy from AIMD simulations.

So far, the experimental results strongly suggest plastic behavior of γ-Na_4_P_2_S_6_, but do not yet distinguish a dynamic versus static nature of the rotational disorder of the P_2_S_6_^4–^ anions. Therefore, the atomic motions of Na^+^ and P_2_S_6_^4–^ were tested using ab initio molecular dynamics (AIMD) simulations, with the PDF-derived 2 × 2 × 2 model as a starting point for optimization and subsequent MD calculations at different sampling temperatures (600–1200 K). The average mean-squared displacement of the atoms of Figure 4b supports the significant mobility of all Na^+^, while P and S atoms do not undergo long-range motion. The calculated activation energy for Na^+^ migration above 800 K (the MD calculation temperature) is 223 meV, though it is again much higher than the experimentally obtained activation barrier (89(18) meV). For the 600 K calculation, the Na^+^ diffusivity is many orders of magnitude smaller.

To determine the possibility for dynamic rotational motion of P_2_S_6_^4–^ anions, the P–P bond orientations were tracked during the AIMD simulations. A detailed discussion about the analysis of the AIMD simulation can be found in the Supporting Information. The orientation of individual P–P bonds within the cubic cell can be expressed in terms of azimuthal (ϕ) and polar (θ) angles as depicted in Figure 5a. Starting from P–P orientations marked as orange dots, the orientation of all 16 P_2_S_6_^4–^ anions were tracked throughout the AIMD simulation and shown as a 2D histogram in Figure 5b. The polar coordinate system allows for the division of the observed eight clusters into eight octants as depicted at the bottom right in Figure 5a. Because the anions rotate about their center of mass, rather than the P atom fixed at the origin, octants related by inversion (e.g., octants II and VIII) signify the same orientation of the P–P handle with respect to the crystal lattice (but with inverted P atoms). Thus, the ensuing population of these initially empty octants indicates that significant reorientation of individual anions occurs. The four orientational groupings point approximately along the body diagonals of the cell. Notably, the population density of the 2D histogram at 1000 K is not uniform after the simulation time of 300 ps. With higher simulation temperature the population density approaches a more uniform distribution of orientations, as depicted in Figure S9. Longer simulation runs would result in a similar picture, i.e., a more equal distribution of orientations.

**Figure 5:**
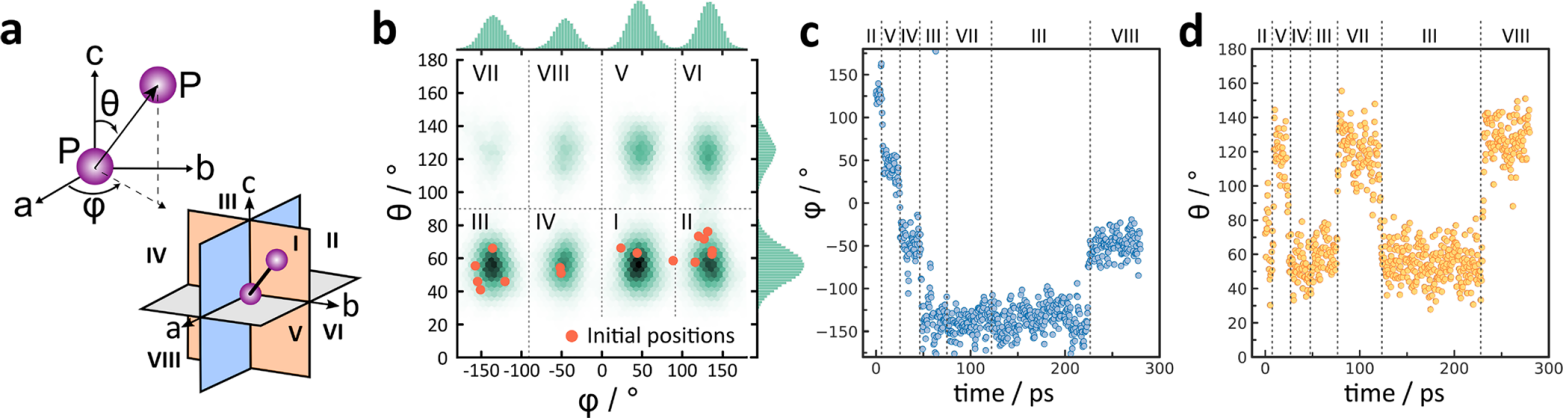
(a) Scheme of the definition of the azimuthal angle (ϕ) and polar angle (θ) with respect to the P–P handle and scheme of the P–P orientation in Cartesian octants. (b) Orientation heat map of all P_2_S_6_^4–^ in a 2 × 2 × 2 cell at 1000 K in a 300 ps simulation. (c) ϕ and (d) θ as a function of time of an exemplary P_2_S_6_^4–^ at 1000 K.

Panels c and d of Figure 5 illustrate the ϕ and θ angles of an exemplary anion from the AIMD simulations at 1000 K and enable the derivation of reorientation times of 20–100 ps. The time evolution of the azimuthal and polar angles of four other anions are illustrated in Figure S10. Here, a direct comparison to plastic phases of sodium *closo*-borane Na_2_B_12_H_12_ can be made: just above the first-order transition from the low-*T* monoclinic to the high-*T* cubic phase near 520 K, the reorientational jump rate, τ^–1^, derived from NMR experiments, is 10^11^ Hz (equal to 10 ps),^27^ a value comparable to the time between P_2_S_6_^4–^ reorientations, although the different shape and size of the anions should be taken into account. Meanwhile, the Na^+^ short-ranged hopping time was calculated to be ∼0.6 ps at 1000 K (see SI Figure S13), 2 orders of magnitude faster than the anion motion.

Another degree of freedom for the anion is conformational freedom or rotation around the PS_3_–PS_3_ bond, similar to ethane and its derivatives. This is measured as the dihedral angle spanned by S–P–P–S. The AIMD simulations suggest that the P–P bond rotation happens on a time scale similar to that of the directional reorientation of the entire anion. We refer to the Supporting Information for a more detailed analysis, including figures depicting the time and population evolution of the dihedral angle.

To summarize, while showing dynamic disorder, the orientation distribution after 300 ps suggests that the anions prefer to point roughly along the four body diagonals of their respective unit cells. This is in agreement with the on-average (*Im*3̅*m*) structure obtained from Rietveld refinement, where the P–P bonds of all orientations on the lattice point overlap to form a roughly cubic distribution that is co-oriented with the crystallographic cell (see the Supporting Information). The results suggest that dynamic reorientation of the P_2_S_6_^4–^ units occurs, consisting primarily of hops between the four body diagonal orientations of the cubic lattice. The frequency of the P_2_S_6_^4–^ reorientation increases with the simulation temperature. Further analysis of this behavior suggests that reorientation of one anion does not necessarily induce a change in its neighbors’ orientations.

In this work we have identified a new high-temperature phase transition at 580 °C in the thiophosphate Na^+^ conductor Na_4_P_2_S_6_. The phase transition is accompanied by a large latent heat and a change in crystal structure (from monoclinic *C*2/*m* to cubic *Im*3̅*m*), as well as a jump in sodium ion transport properties. A structure model with P_2_S_6_^4–^ anions occupying the bcc lattice points was derived from pair distribution function analysis. The PDF structure model was used to simulate cation and anion dynamics with *ab initio* molecular dynamics. We demonstrate that while sodium shows a high diffusion coefficient in the AIMD simulation, the P_2_S_6_^4–^ anions form a translationally fixed sublattice characterized by orientational disorder and the ability to dynamically reorient, primarily between the four body diagonal directions. Na_4_P_2_S_6_ is malleable at high temperature and mechanically deforms under even slight compressive stress: a property in solid ion conductors that is unique to plastic phases. Altogether, the evidence suggests that γ-Na_4_P_2_S_6_ is a new member of the plastic ionic conductors.

Despite the advantageous mechanical and conduction properties of plastic ion conductors, the temperatures needed to stabilize these phases are still far too high for operation in solid-state batteries. A key goal in solid-state battery chemistry is thus to stabilize such plastic phases at close to ambient temperature. Thus, further search and a detailed understanding of the underlying structure–property relationships of such phases will be necessary to identify plastic phases with lower transition temperatures.

## Experimental Methods

### Preparation of Na_4_P_2_S_6_

Na_4_P_2_S_6_ can be prepared using two different synthesis routes. A solid-state reaction as described in ref (10) yields a very crystalline powder from which also single crystals can be extracted. Further, the solution synthesis via the hydrated compound Na_4_P_2_S_6_·6H_2_O as in ref (9) gives a powder product with stacking faults. The latter anneals above 500 °C to a product very similar to the solid-state material. The structural differences are discussed in detail in ref (11). The β → γ phase transition is not affected by the Na_4_P_2_S_6_ synthesis.

### Variable-Temperature Powder X-ray Diffraction

Variable-temperature powder X-ray diffraction (PXRD) patterns were measured using a STOE StadiP diffractometer (Mo-Kα_1_ radiation (λ = 0.7093 Å), curved germanium (111) monochromator, DECTRIS Mythen2R 1K detector) with a STOE capillary furnace in Debye–Scherrer geometry. Fine powdered samples were filled in quartz glass capillaries of 0.5 mm diameter by Hilgenberg and sealed under argon. Data collection was done in the range from 2° to 62° 2θ with a step size of 0.015° in the temperature range of 510, 650, and 440 °C in steps of 5 K around the immediate β → γ phase transition and 10 K for more distant temperatures. The indexing of the powder patterns was performed with Jana2006(45) in space groups *C*/2*m* and Im3̅*m* for β- and γ-Na_4_P_2_S_6_, respectively. Errors of the results of the Rietveld refinements are specified as 3σ.

### Total Scattering Measurements

Total scattering measurements were carried out using beamline P02.1 at PETRA III of the Deutsches Elektronen-Synchrotron (DESY). Data were collected in rapid acquisition mode^46^ with a large-area 2D PerkinElmer detector (2048 × 2048 pixels, 200 × 200 μm^2^ each) and sample-to-detector distance of 311.36 mm. The incident energy of the X-rays was 59.772 keV (λ = 0.20743 Å). Samples were loaded into 0.7 mm diameter quartz glass capillaries and measured at roughly 30 and 100–650 °C in increments of 50 °C. An empty capillary was measured to account for the background, and a LaB_6_ standard was measured at room temperature for calibration of the setup. Sample temperature was controlled using a custom ceramic heater operated using a Eurotherm 2408 temperature controller. Calibration, polarization correction, and azimuthal integration to 1D diffraction patterns were performed using the software pyFAI.^47^ Further correction, background subtraction, and normalization of the 1D diffraction intensities were carried out to obtain the total scattering structure function, *F*(*Q*), which was Fourier transformed to the PDF, and *G*(*r*), using PDFgetX3 within xPDFsuite.^48,49^ The maximum value of the momentum transfer, *Q*, used in the Fourier transform was 19.5 Å^–1^. Additional total scattering measurements were performed using a Stoe Stadi-P diffractometer with Ag Kα_1_ radiation (λ = 0.55941 Å), a Ge(111) Johann monochromator, and an array of three DECTRIS Mythen 1K detectors measured at two different positions in Debye–Scherrer geometry. The temperature was controlled using a hot air blower from FMB Oxford. Data were directly corrected for the 2θ offset of the instrument, polarization, and background scattering.

### Pair Distribution Function and Rietveld Refinements

Pair distribution function (PDF) refinements were carried out using TOPAS Academic v6.^50,51^ In all refinements, the P_2_S_6_^4–^ anions were set up as rigid bodies defined by the P–P and P–S bond lengths and P–P–S bond angle. Anion positions were fixed at the lattice points but allowed to rotate freely about their center of mass. When added, Na atom positions were allowed to be refined with antibump constraints set to prevent unrealistic interatomic distances. PDF refinements were performed in *P*1 symmetry with either two separate anions (single-cell model) refined over 1.5–7 Å, or 16 separate anions (supercell model) refined over 1.5–16 Å. The damping of the experimental signal due to instrumental resolution and effects of data truncation were accounted for and fixed, and the lattice parameter and scale factor were refined. *B*_iso_ was fixed to 2.0 Å^2^ for S and P atoms, and separate intramolecular pair line widths were described with five parameters corresponding to P–P, bonding P–S, adjacent S–S, nonbonding P–S, and opposing S–S, to account for intra-anion disorder.^42^ Since Na atoms only show correlation at short distances, the partial pair widths were described using an empirical function (*x*_1_ + *x*_2_*r* + *x*_3_*r*^2^) that allows for high correlation with neighbor atoms at short distances and progression to low structural correlation at higher distances.

Rietveld refinements^52,53^ were performed with the *Im*3̅*m* model refined over a range of 4–28° 2θ. The background was described using Chebychev polynomials of 19th order. The Lorentz polarization factor was set to 9.825. A zero-error correction was refined to correct for detector offset, and a simple axial model was used to describe the instrumental peak shape along with Gaussian and Lorentzian components for crystallite size and strain broadening. The lattice parameter, global scaling factor, and two separate isotropic displacement parameters for Na and S/P were additionally refined. Convergence of the reciprocal- and real-space refinements was achieved using the global optimization method of simulated annealing in real space.^54^

### Differential Scanning Calorimetry

For differential scanning calorimetry (DSC) measurements, fine powdered samples of 50 mg each were sealed in quartz ampoules (6 mm diameter, 10–15 mm in height) under vacuum. For improved heat flow, ampoules with a flat bottom were used. DSC measurements were performed on a NETZSCH STA 449 F5 Jupiter in a temperature range of 30–850 °C. The heating rate for all segments was set to 20 K min^–1^. This rate is a good trade-off between measurement stability (rate linearity) and sensitivity. Data evaluation was performed using the NETZSCH software package Proteus.

### Electrochemical Impedance Spectroscopy

Electrochemical impedance spectroscopy (EIS) measurements were performed using a NOVOCONTROL Technologies Alpha-A analyzer. For sample preparation a fine powder of Na_4_P_2_S_6_ (ca. 70 mg) was pressed uniaxillly at 1 GPa into pellets of 6 mm diameter. Both sides of the pellets were sputtered with platinum to ensure electrical contact during the high-temperature impedance measurement. Pellets were placed between two lithium-ion blocking platinum electrodes and loaded onto an in-house-built cell. To prevent the deformation of the pellet due to mechanical softening in the γ phase, the pellet was placed in a hole in a ceramic spacer of 1 mm height. This has proven to give reproducible results that are only affected by sample decomposition and corrosion of the electrode by sulfur at high temperature. The equilibration time prior to each impedance measurement at the programmed temperature steps was set to 15 min. A short equilibration time translates into a shorter time the sample is exposed to high temperatures. This was necessary as the sample decomposes within a day at 600 °C or above. On the other hand, short equilibration times bear the difficulty of measuring in a non-equilibrium state that can result in visible drifts in long impedance measurements. Hence, the spectra were recorded in a frequency range of 1 MHz to 10 Hz and with an applied voltage of *V*_RMS_ = 100 mV. The spectra were recorded between 500 and 640 °C. Data treatment and evaluation was performed using the rhd instruments software package RelaxIS 3. To check data reliability Kramers–Kronig relation tests were performed prior to fitting. Fitting the impedance spectra to equivalent circuits was done by weighting the data points proportionally. Given error bars stem from error propagation of uncertainties in pellet geometric area, pellet thickness, and applied temperature as well as errors in resistance obtained by equivalent circuit fitting.

### Raman Spectroscopy

Temperature-dependent Raman spectra were recorded using a Jobin Yvon Type 010 labram single grating spectrometer equipped with a double super razor edge filter. Data collection was performed with a Peltier cooled CCD camera (spectral resolution: 1 cm^–1^).

### Computational Methods

DFT simulations were performed using the Vienna ab initio Simulation Package.^55,56^ The unknown exchange correlation energy in DFT was approximated by the generalized gradient approximation,^57^ and van der Waals forces were captured using the Grimme scheme.^58^ Valence electrons were expanded as plane-waves with a maximum kinetic energy of 520 eV, and DFT total energies integrated at the Γ-point. Core electrons were described with projected augmented wave potentials,^59,60^ treating the following electrons explicitly: Na (2s^1^), S (3*s*^2^3*p*^4^) and P (3*s*^2^3*p*^3^).

Starting from the PDF-derived crystal structures of γ-Na_4_P_2_S_6_ (2 × 2 × 2 cell), all P_2_S_6_^4–^ units were oriented along the ⟨111⟩ directions and being perpendicular with each other in the (100) and (010) planes. Then a structure optimization was performed until the interatomic forces are less than 0.01 eV/Å while keeping the cell volume fixed to that derived from the temperature-dependent diffraction experiments.

Using the DFT-optimized γ-Na_4_P_2_S_6_ model, canonical (*NVT*) ensemble *ab initio* molecular dynamics (AIMD) simulations were performed at selected temperatures of 600, 800, 900, 1000, and 1200 K, respectively. The *NVT* ensemble was achived via a Nosé–Hoover thermostat^61,62^ with a 2 fs time step. The simulations were initialized at 100 K, and temperatures were ramped to target values within 2 ps. MD runs were performed with a production time of 600 ps (for 600 and 800 K) and 300 ps (for 1000 and 1200 K). Trajectories were collected for the data analysis excluding the initial period of 20 ps for equilibration.

## Supporting Information Available

Additional details on diffraction experiments, Raman spectroscopy, pair distribution function analysis, impedance spectroscopy, photographs of impedance samples, and AIMD simulation results (PDF)
Single-cell γ-Na_4_P_2_S_6_ structure obtained from PDF refinement (CIF)
2 × 2 × 2 supercell γ-Na_4_P_2_S_6_ structure obtained from PDF refinement (CIF)
γ-Na_4_P_2_S_6_ structure obtained from Rietveld refinement (CIF)

## Supplementary Material

nz1c02815_si_001.pdf

nz1c02815_si_002.cif

nz1c02815_si_003.cif

nz1c02815_si_004.cif
