# Supplementary material for: Superionic Conduction in the Plastic Crystal Polymorph of Na4P2S6
Source: ACS Energy Lett. 2022 Mar 22;7(4):1403–11. doi: 10.1021/acsenergylett.1c02815 (PMC9008513; doi:10.1021/acsenergylett.1c02815)
Supplement: Supplementary file 1 — nz1c02815_si_001.pdf [file nz1c02815_si_001.pdf]

Supporting Information: Superionic Conduction in the Plastic Crystal Polymorph of $Na_4P_2S_6$

Tanja Scholz,[†,‖] Christian Schneider,[†,‖] Maxwell W. Terban,[†] Zeyu Deng,[‡] Roland Eger,[†] Martin Etter,[¶] Robert E. Dinnebier,[†] Pieremanuele Canepa,[*,‡,⊥] and Bettina V. Lotsch[*,§,†]

†*Max Planck Institute for Solid State Research, Heisenbergstraße 1, 70569 Stuttgart, Germany*

‡*Department of Materials Science and Engineering, National University of Singapore, 9 Engineering Drive 1, 117575, Singapore*

¶*German electron synchrotron (DESY), Notkestraße 85, Hamburg, 22607, Germany*

§*LMU Munich, Butenandstraße 5-13, 81377 Munich, Germany*

‖*Contributed equally to this work.*

⊥*Department of Chemical and Biomolecular Engineering, National University of Singapore, Engineering Drive 4, 117585, Singapore*

E-mail: pcanepa@nus.edu.sg; b.lotsch@fkf.mpg.de

Variable Temperature Powder X-Ray Diffraction

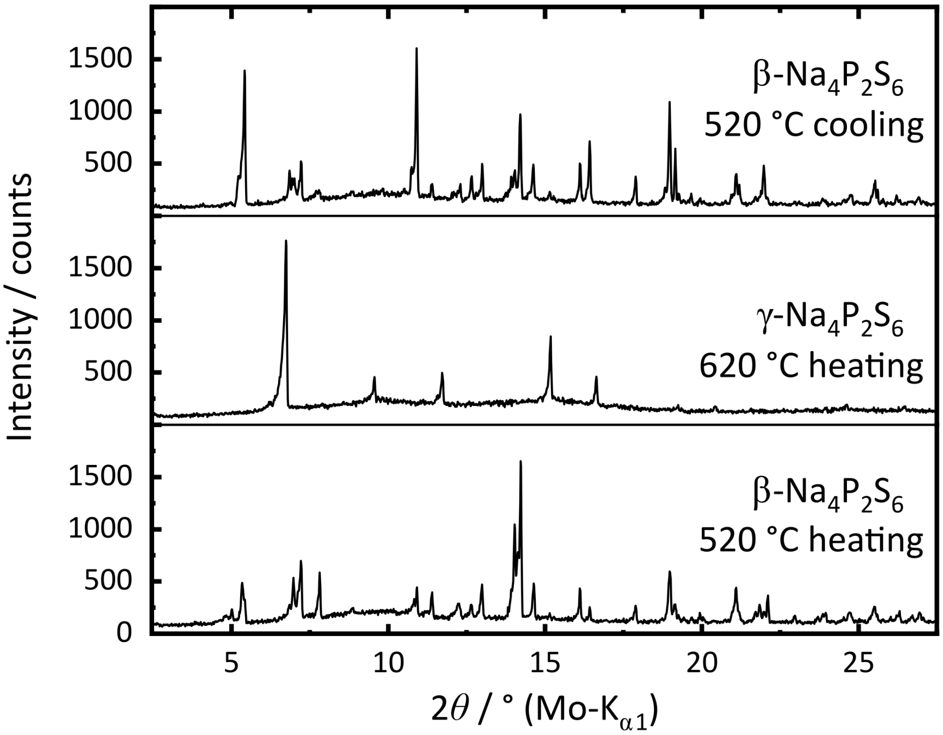

Figure S1: Powder X-Ray diffraction pattern of β-$Na_4P_2S_6$ at 520 °C upon heating and cooling, and of γ-$Na_4P_2S_6$ at 620 °C. The patterns were extracted from the measurement depicted in Figure 1. One hour measurement time per pattern; about 51 hours in total.

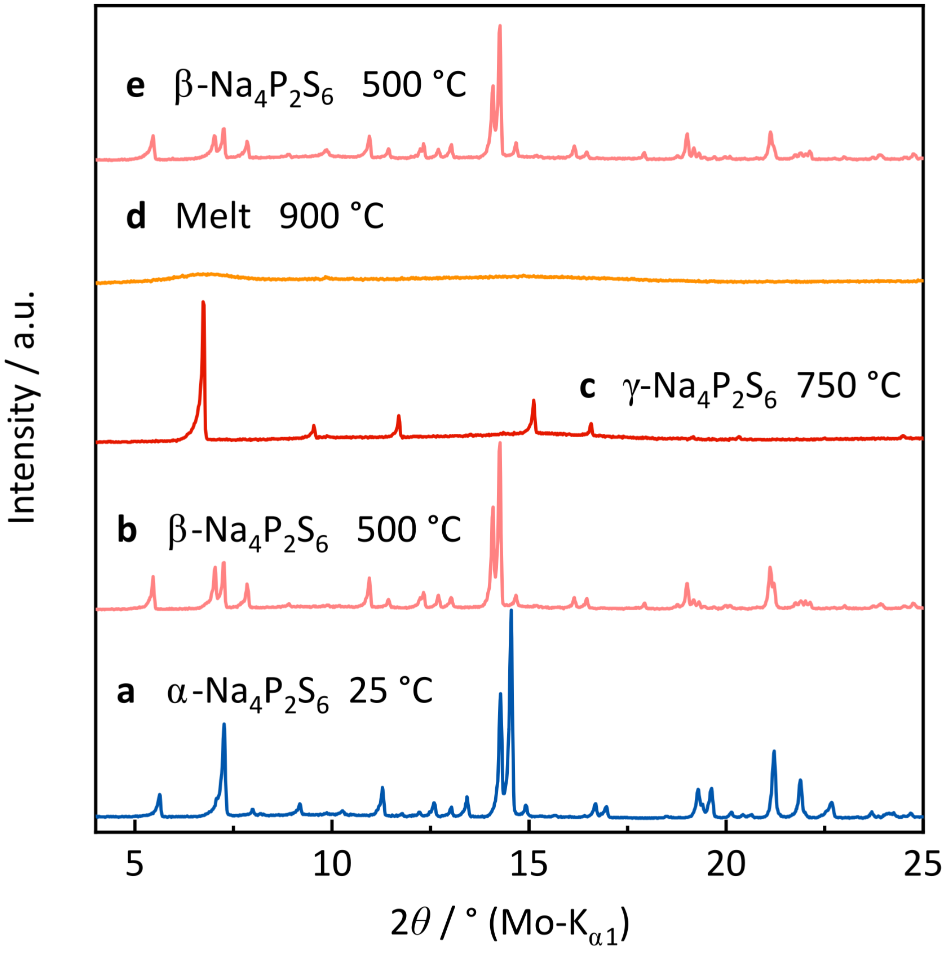

Figure S2: Powder X-Ray diffraction pattern of **a** α-$Na_4P_2S_6$ at 25 °C, **b** β-$Na_4P_2S_6$ at 500 °C, **c** γ-$Na_4P_2S_6$ at 700 °C, **d** molten $Na_4P_2S_6$ at 900 °C, and **e** β-$Na_4P_2S_6$ at 500 °C after crystallizing from the melt. One hour measurement time per pattern; about 6 hours in total.

Table S1: Crystallographic data and refinement details. Estimated standard deviations as obtained from the Rietveld refinements are given in parentheses.

	γ-$Na_4P_2S_6$
Temperature ϑ (°C)	650
Formula weight M ($g\,mol^{-1}$)	692.593
Crystal system	cubic
Space group	$Im\bar{3}m$ (no. 229)
Lattice parameter a (Å)	8.4851(5)
Volume V ($Å^3$)	610.91(10)
Formula units Z	2
Crystallographic density ρ ($g\,cm^{-3}$)	1.8826(3)
X-ray radiation	Ag $K\alpha_1$
2θ range (°)	4–28
No. of refined parameters	37
R_{exp} (%)	1.90
R_p (%)	2.10
R_{wp} (%)	2.77
R_{Bragg} (%)	0.36

Table S2: Atomic positions and displacement parameters of γ-$Na_4P_2S_6$ at 650 °C. Estimated standard deviations as obtained from the Rietveld refinement are given in parentheses.

Atom	Wyckoff position	x	y	z	Occupancy	B_{iso} ($Å^2$)
Na1	96l	0.729(4)	0.271(4)	0.151(4)	1/24	6(2)
Na2	96l	0.731(6)	0.455(6)	0.140(2)	1/24	6(2)
P1	96l	-0.090(6)	0.057(5)	-0.079(5)	1/48	11.5(4)
P2	96l	0.090(6)	-0.057(5)	0.079(5)	1/48	11.5(4)
S1	96l	-0.099(11)	0.287(4)	-0.016(16)	1/48	11.5(4)
S2	96l	-0.011(16)	0.026(16)	-0.3024(15)	1/48	11.5(4)
S3	96l	-0.2956(17)	-0.058(11)	-0.038(15)	1/48	11.5(4)
S4	96l	0.011(16)	-0.026(16)	0.3024(15)	1/48	11.5(4)
S5	96l	0.099(11)	-0.287(4)	0.016(16)	1/48	11.5(4)
S6	96l	0.2956(17)	0.058(11)	0.038(15)	1/48	11.5(4)

Raman Spectroscopy

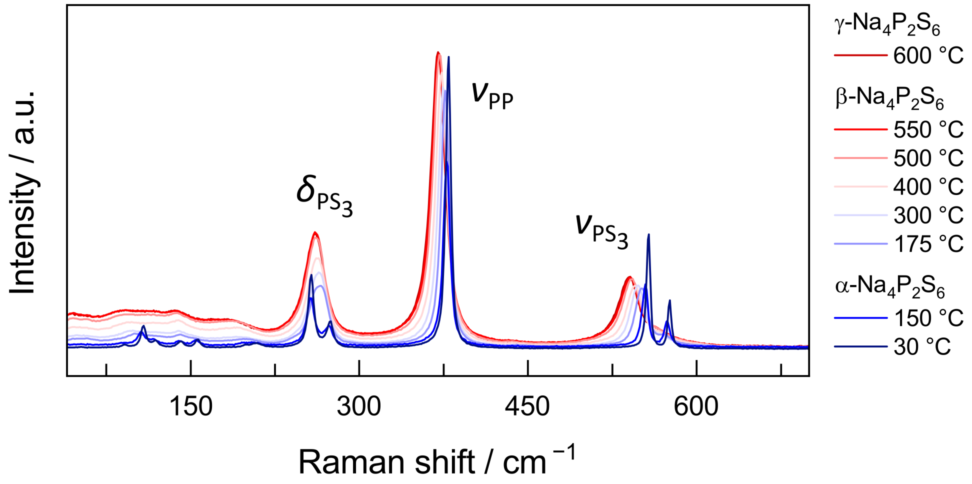

Figure S3: Raman spectra of α-, β-, and γ-$Na_4P_2S_6$.

Pair Distribution Function Analysis

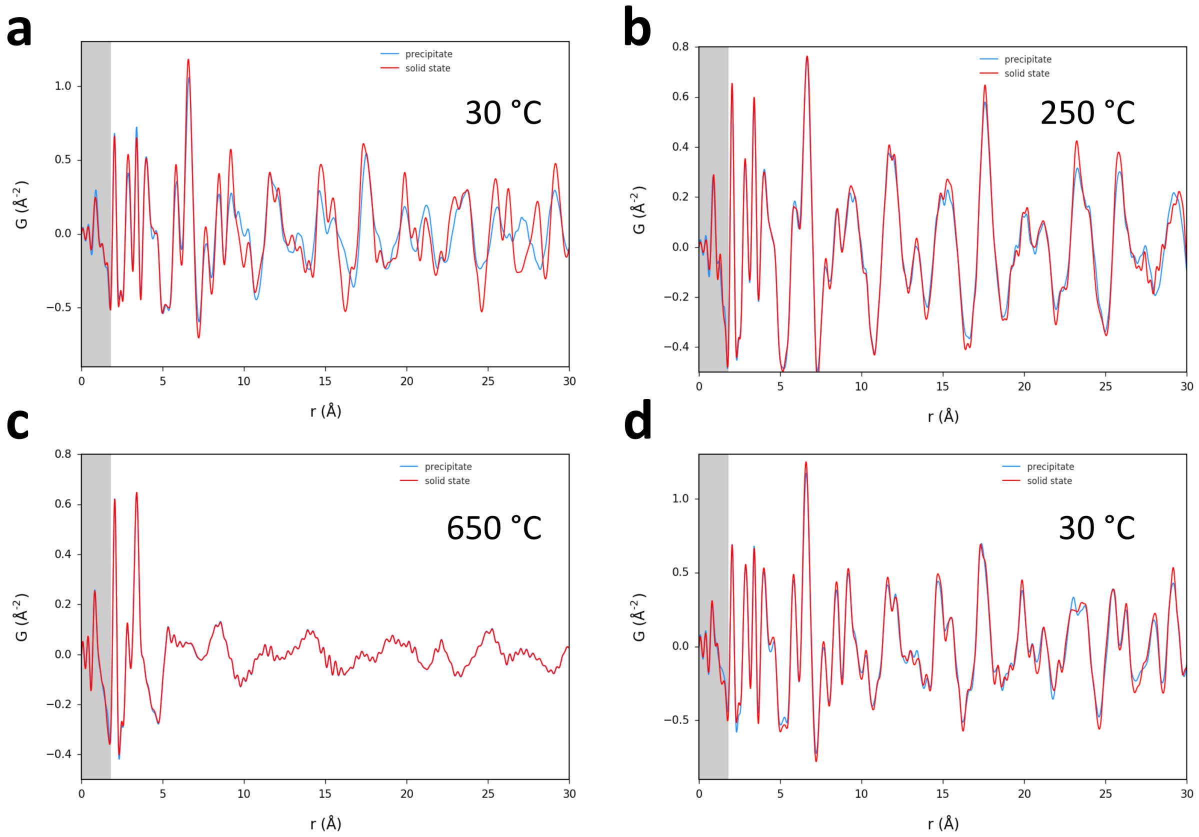

Figure S4: PDFs of solid-state synthesized and precipitated $Na_4P_2S_6$ measured **a** on the pristine samples at 30 °C, **b** in the β-phase regime at 250 °C, **c** in the γ-phase regime at 650 °C, and after the high temperature measurements at 30 °C. The grey shaded region represents the unphysical interatomic distance range that is more highly affected by systematic errors from data processing.

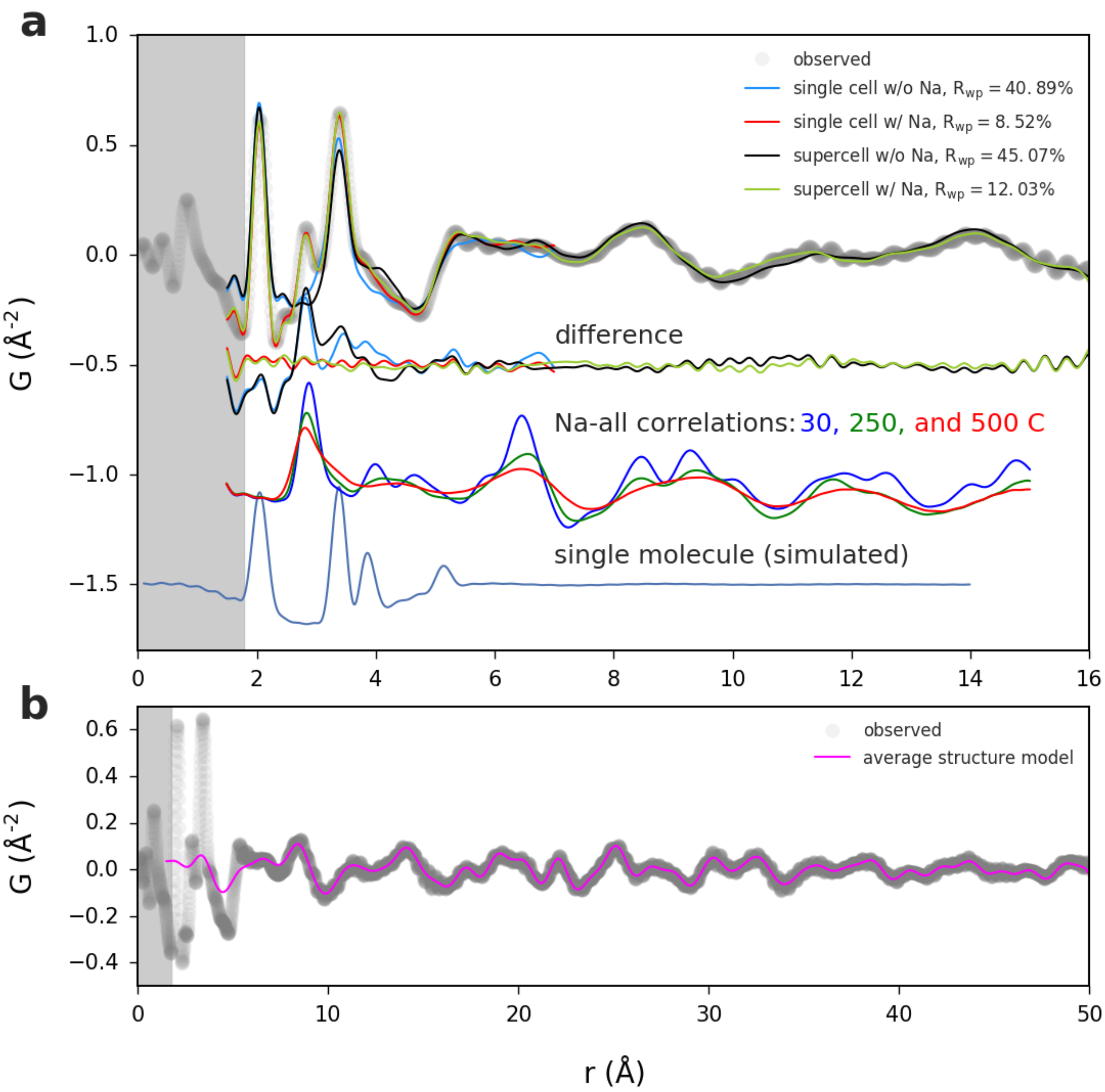

Figure S5: **a** Comparison of the measured and fitted PDFs of the single cell and super-cell structure models of γ-$Na_4P_2S_6$ (constructed with $P1$ symmetry), with and without sodium contribution. For reference, the partial PDFs between Na and all other atoms were extracted from fits to the lower temperature data to index the Na-S distances around ∼3 Å. The PDF simulated for a single $P_2S_6{}^{4-}$ anion is also shown. **b** A fit of the average structure with overlapping anion orientations ($Im\bar{3}m$) obtained from Rietveld refinement to the high-r region of the PDF. The grey shaded region represents the unphysical interatomic distance range that is more highly affected by systematic errors from data processing.

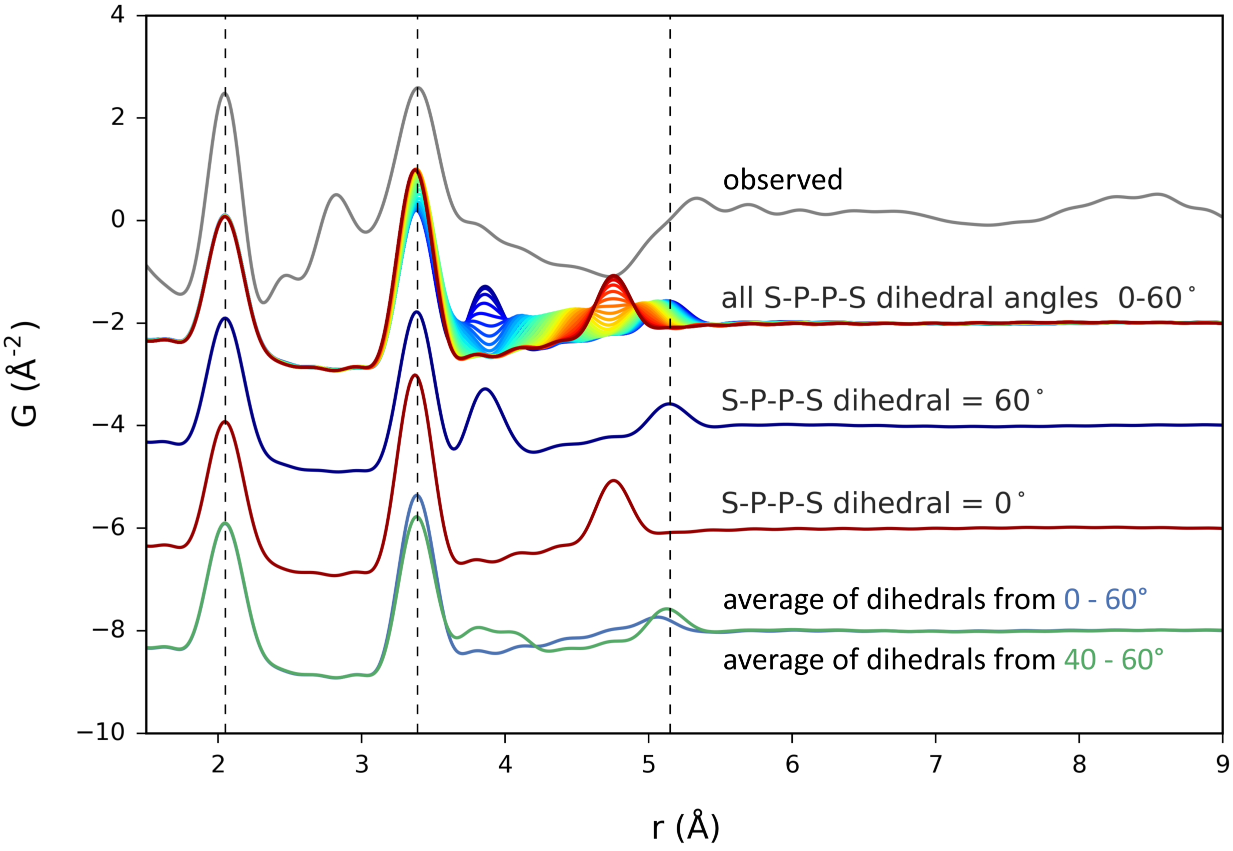

Figure S6: Analysis of the effect of S-P-P-S dihedral angle on the intramolecular PDF signal for a $P_2S_6{}^{4-}$ anion. The PDFs for staggered (60°) and eclipsed (0°) conformations are shown for comparison, along with an overlay of PDFs for all conformations. The lack of the short S-(P-P)-S pair correlation just under 4 Å suggests the possibility for some distribution of the dihedral angles. We show that this peak can be washed out by averaging all possible conformations, but also more simply, by averaging conformations over a range of just 20° around the preferred, staggered conformation. The long S-(P-P)-S peak cannot be distinctly resolved to help further disambiguate these cases.

Impedance Spectroscopy

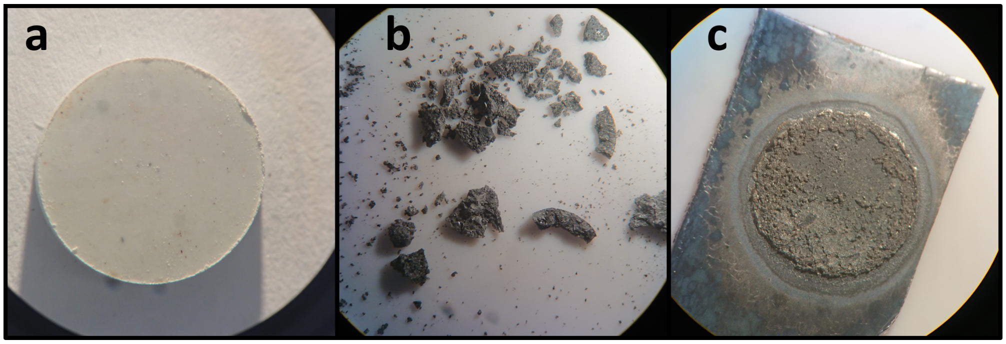

Figure S7: Images of **a** pristine $Na_4P_2S_6$ pellet, **b** broken pieces of a $Na_4P_2S_6$ pellet after high-temperature impedance measurement, and **c** the Platinum electrode after being in contact with $Na_4P_2S_6$ at 640 °C.

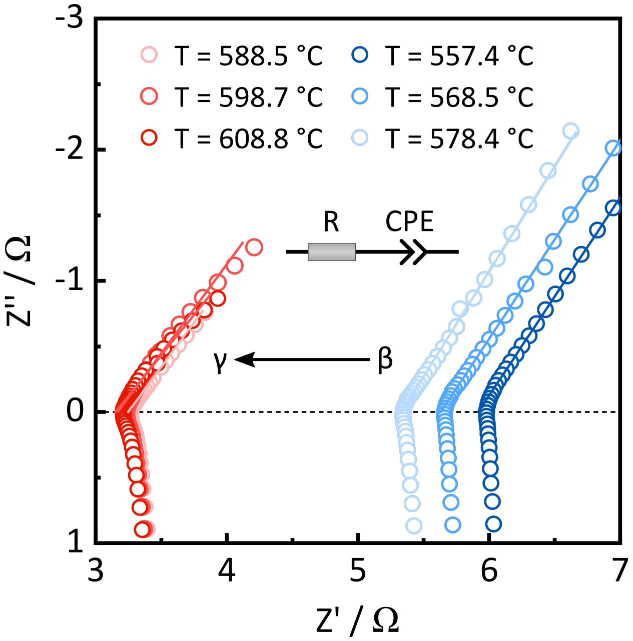

Figure S8: Exemplary Nyquist plots of high temperature impedance measurements (hollow circles) and equivalent circuits fits (lines) at six selected temperatures.

Experimental difficulties and aggressive nature of $Na_4P_2S_6$

In this section we discuss the experimental difficulties we faced while characterizing γ-$Na_4P_2S_6$ with high-temperature diffraction, DSC and impedance spectroscopy. The following

points summarize the experiments in which we noticed the chemically aggressive nature of γ-$Na_4P_2S_6$ and describe the visual changes we observed:

- High-temperature diffraction: Starting with white to light-gray powder, we noticed a darkening to gray in every HT (diffraction) experiment we performed. The color change was often accompanied with increasing issues in the mechanical stability of the capillary with increasing measurement time. Longer experiments resulted in capillaries that were difficult to unload from the furnace. Hair-cracks probably formed over time and resulted in a not airtight seal of the capillary. Sulfur loss and/or decomposition could be the cause of the extreme brittleness of the glass capillary. Shorter experiments were less affected.

- Preferred orientation: In some longer HT PXRD experiments we noticed a change of the reflection intensities before and after heating to the γ-phase (see Figure S1). Another, shorter HT PXRD experiment does not show any of this change (compare Figure S2b and e). We suspect the before mentioned capillary issue to be one possible explanation of this effect. Another explanation could the the formation of preferred orientation of crystallites during a rather slow cool-down. We fitted the HT PXRD data with higher order spherical harmonics to correct for preferred orientation. However, form fitting, we could not tell if preferred orientation or a compositional change by sulfur loss and/or decomposition leads to the change in intensity. It is plausible that both effects contribute to the observation.

- Differential scanning calorimetry: To test the thermal stability of $Na_4P_2S_6$ pellets, we performed a simple thermogravimetric experiment. We observed a slow, monotonic mass loss at 650 °C (12 hours). At the top of the furnace sulfur vapor condensed. Thus, we suspect that $Na_4P_2S_6$ slowly, but constantly, evaporates sulfur if the material is not contained in a closed vessel. Additionally, we noticed that the Al_2O_3 crucibles started to become porous and allowed $Na_4P_2S_6$ to leak and consequently corrode the

platinum wires of the thermocouple.

- High-temperature impedance spectroscopy: As shown in image S7c, the platinum electrodes severely suffered from corrosion. After the experiment the electrodes needed to be replaced and the connecting wire had to be cut to a shorter length, since they started to corrode at the jointed end. We suspect that sulfur vapor attacks the platinum components of the setup and consequently forms PtS or PtS_2, materials with a small bandgaps of 0.471 eV and 1.444 eV.[S1]

Additional Information on the $P_2S_6{}^{4-}$ Dynamic Disorder

The individual $P_2S_6{}^{4-}$ anions were placed into a reference frame with one P atom fixed to the origin of a Cartesian coordinate system that aligns with the unit cell axes at all times (see Figure 5a, top left). Then, the orientations were expressed in terms of polar coordinates as a function of azimuthal (ϕ) and polar (θ) angles. Visually, the chosen origin divides the possible orientations into eight octants as depicted in Figure 5a. Because the anions rotate about their center of mass, rather than the P atom fixed at the origin of the reference frame, octants related by inversion signify the same orientation of the P–P handle with respect to the crystal lattice (but with inverted P atoms). Note that with each simulation frame (*i.e.* reorientational motion of the $P_2S_6{}^{4-}$ anion), the reference frame origin changes with respect to the crystal lattice, such that the center of mass of the P–P handle remains translationally fixed. Thus, the ensuing population of these initially empty octants indicates that significant reorientation of individual anions occurs.

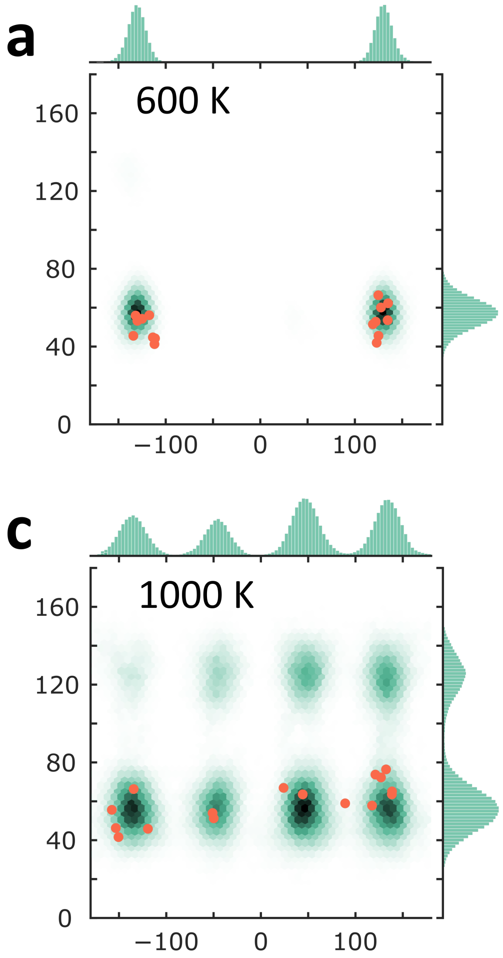

Figure S9: Orientation heatmap of all $P_2S_6^{4-}$ in a 2×2×2 supercell at **a** 600 K, **b** 800 K (both 600 ps calculations), **c** 1000 K, and **d** 1200 K (both 300 ps calculations). Initial positions are depicted in orange.

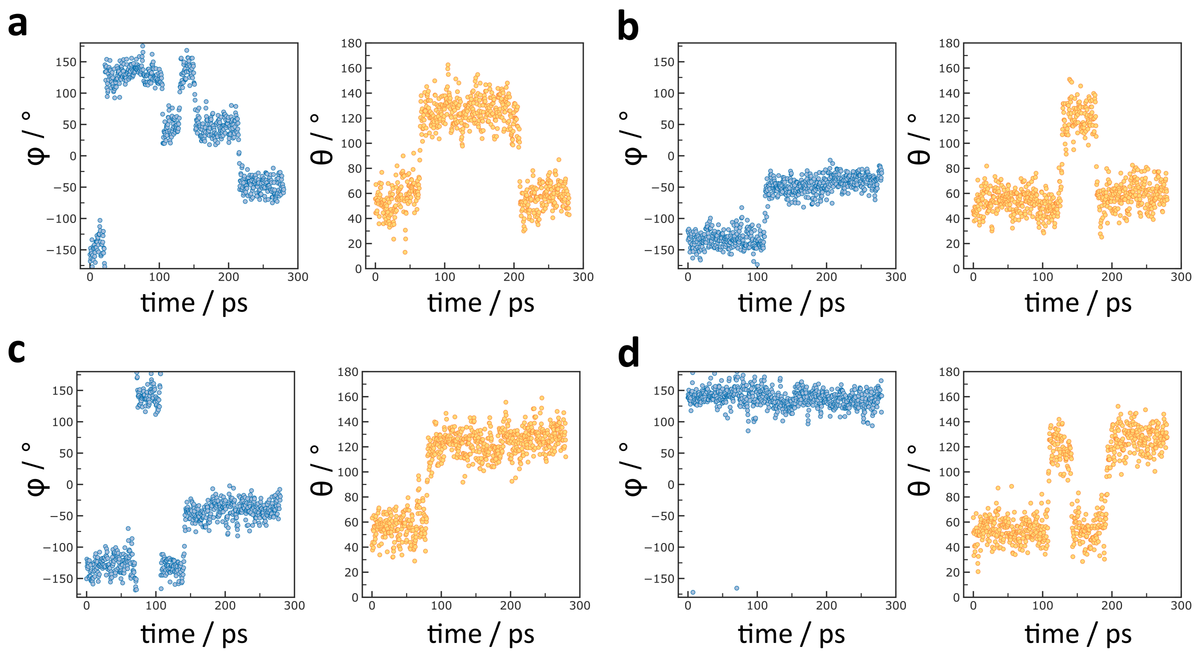

Figure S10: Azimuthal angle (ϕ) and polar angle (θ) as a function of time of four $P_2S_6{}^{4-}$ at 1000 K.

Analysis of the S–P–P–S Dihedral Angle Dynamics

The conformational freedom of the $P_2S_6{}^{4-}$ anions was investigated. Rotation around the PS_3–PS_3 bond is possible (see Figure S11). The initial anions had a *gauche* conformation ($|\sphericalangle| = 60°$) as observed in the α and β crystal structures. To capture the evolution of the $P_2S_6{}^{4-}$ dihedral angle for the ensemble of anions, we counted the number of dihedral angles for different time scales. The histogram plots depicted in Figure S11b,b,e and f show an increase in the *anti* ($|\sphericalangle| = 180°$), and Figure S12 show the time evolution of the dihedral angle of three randomly chosen S-P–P-S units. Although rotations around the P–P axis are observed, some anions showed no conformational reorientation on the 300 ps time scale. The likelihood for rotation around the P–P bond of the anions is supported by the lack of sharp correlations between the S-P–P-S atom-pairs in the experimental PDF and the corresponding need for significantly broadening these contributions in fitting the model.

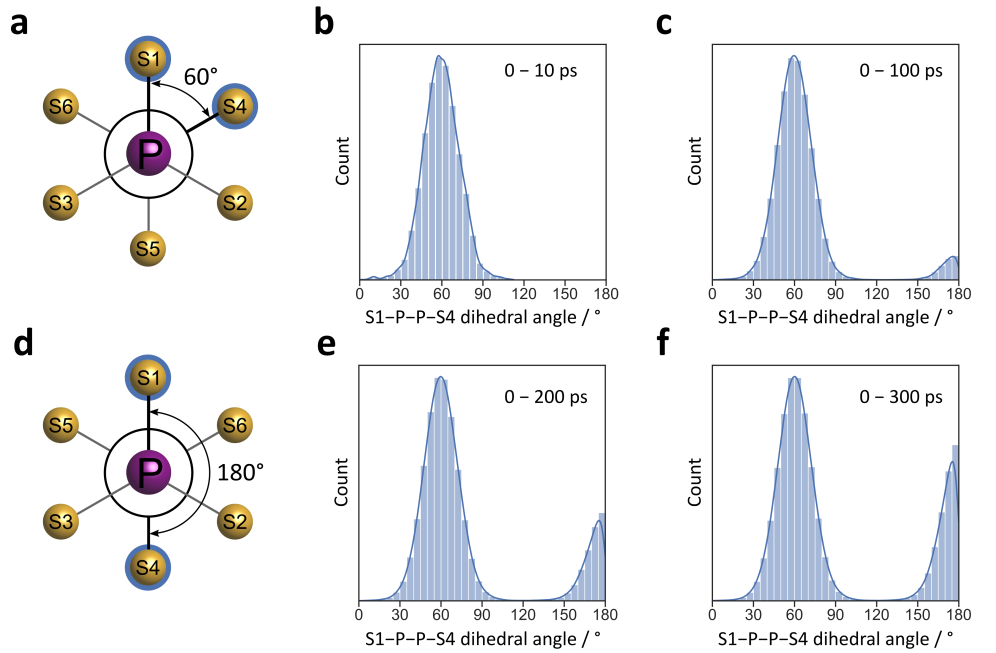

Figure S11: Newman projection of the $P_2S_6{}^{4-}$ anion in the staggered conformation with a dihedral angle of **a** 60° and **d** 180°. Histograms of the dihedral angles of the $P_2S_6{}^{4-}$ anions (defined by two planes spanned by S1–P–P–S4) after the simulation time of **b** 10 ps, **c** 100 ps, **e** 200 ps and **f** 300 ps has elapsed.

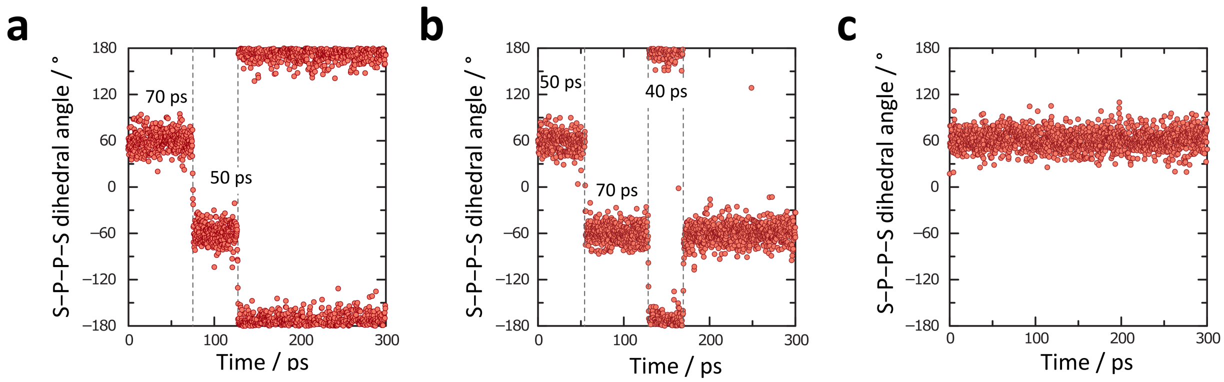

Figure S12: Dihedral angles (S1–P–P–S4, as defined in Figure S8) of three $P_2S_6{}^{4-}$ anions, randomly chosen from the $2 \times 2 \times 2$ supercell, as a function of time (in ps). **a** and **b** depict the diheral rotation of one PS_3 group around the P–P bond, whereas in **c** no rotation is observed in the simulation time of 300 ps (1000 K).

Short-ranged Na^+ Hopping Time Analysis

Na^+ short-ranged hopping time was evaluated by analysing the Na^+ trajectory from AIMD. The simulation cell was divided into $4 \times 4 \times 4$ identical cubic units each with a volume of ~ 76.34 Å^3. A hopping event happens when Na^+ crosses a boundary of these units. The hopping time for each Na^+ starts to count from 0, and it is checked every dt ($dt = 10$ fs in this study) to see whether there is a hopping event. If there is a hopping event, the total hopping time will be recorded and reset to 0. If there is no hopping event within dt, the hopping time of this Na^+ will keep on accumulating until it crosses a boundary. Finally, the hopping time was averaged over all hopping events as well as over all Na^+ at each temperature, which is shown in Figure S13.

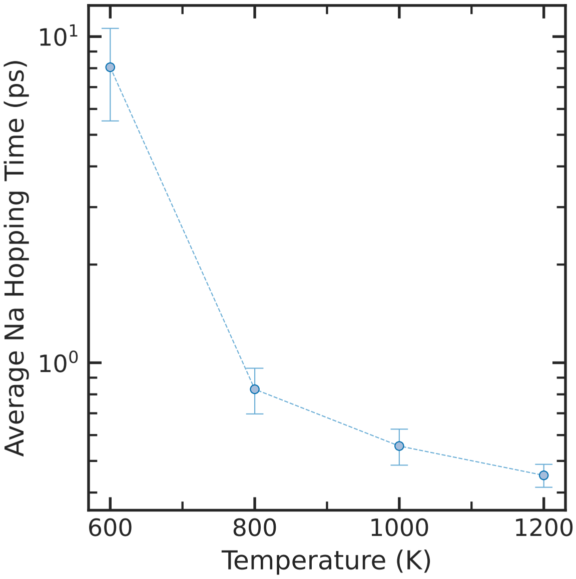

Figure S13: Averaged short-ranged hopping time (in ps) of Na^+ as a function of temperature (in K). Error bar shows the standard deviation between different Na^+.

References

(S1) Jain, A.; Ong, S. P.; Hautier, G.; Chen, W.; Richards, W. D.; Dacek, S.; Cholia, S.; Gunter, D.; Skinner, D.; Ceder, G.; Persson, K. a. The Materials Project: A materials genome approach to accelerating materials innovation. APL Materials **2013**, 1, 011002.
